# Neurological Manifestations of Non-Severe COVID-19—A Multidirectional Approach

**DOI:** 10.3390/brainsci13020355

**Published:** 2023-02-18

**Authors:** Jakub Udzik, Anna Kowalczyk, Aleksandra Waszczyk, Zuzanna Nowaczyk, Andrzej Barczyszyn, Karolina Działa, Maciej Mularczyk, Małgorzata Niekrasz

**Affiliations:** 1 Department of Physiology, Pomeranian Medical University, 70-111 Szczecin, Poland; 2 Department of Neurology and Cerebral Stroke, Samodzielny Publiczny Wojewódzki Szpital Zespolony, 71-455 Szczecin, Poland; 3 Department of Infectious Diseases, Hepatology and Liver Transplantation, Pomeranian Medical University, 71-455 Szczecin, Poland; 4 Neurosurgery Department, Independent Public Clinical Hospital Nr. 1, Pomeranian Medical University, 71-252 Szczecin, Poland; 5 Department of Radiology and Diagnostic Imaging, Samodzielny Publiczny Wojewódzki Szpital Zespolony, 71-455 Szczecin, Poland; 6 Department of Anatomy, Pomeranian Medical University, 70-111 Szczecin, Poland

**Keywords:** COVID-19, SARS-CoV-2 infection, EEG in COVID-19, MRI in COVID-19, COVID-19 symptoms, neuro-COVID-19, brain microhemorrhages

## Abstract

COVID-19 remains a significant clinical issue worldwide, with frequent neurological manifestations. In this study, the authors combine data obtained from the patient’s medical history, physical examinations, and additional test results in the pursuit of any clinically relevant connections. Fifty-eight adult patients hospitalized in the Department of Neurology and Infectiology over a five-months period were retrospectively enrolled in this study. On admission, all patients included in this study were classified as mild or moderate COVID-19 cases, according to the World Health Organization (WHO) guidelines. Laboratory tests, Electroencephalography (EEG), and Magnetic Resonance Imaging (MRI) were performed. There was no statistically significant difference in the Neutrophil–Lymphocyte Ratio (NLR), C-reactive protein (CRP), and Interleukin 6 (IL-6) in patients who reported to the hospital within a week from the symptoms’ onset and in those who reported later. In total, 49.06% of patients with eligible EEG recordings presented abnormal brain activity, while 27.59% of the study population had COVID-19-associated MRI findings. EEG and MRI abnormality occurrence did not correlate with the incidence of mild neurological symptoms (headache, olfactory, and gustatory disorders) of the SARS-CoV-2 infection. In three patients in this study population, unprovoked generalized epileptic seizures occurred for the first time in their life. Non-severe SARS-CoV-2 infection causes functional and structural abnormalities within the central nervous system. Brain microhemorrhages are frequently present in non-severe COVID-19 patients. There is no significant association between mild neurological symptoms of COVID-19 and additional test abnormalities. The time from SARS-CoV-2 infection’s onset to hospital admission does not seem to influence the prognostic value of CRP, IL-6, and NLR in non-severe COVID-19. Mild-to-moderate SARS-CoV-2 infection can be a trigger factor for epilepsy and epileptic seizures.

## 1. Introduction

As COVID-19 remains an important clinical issue worldwide, more and more data emerge regarding the pathophysiology and possible clinical manifestations of the SARS-CoV-2 infection. Discovery of the ACE-2 receptor’s role in the virus’s life cycle enabled physicians to understand the variety of symptoms associated with this disease [1]. This includes a wide range of neurological disorders observed in COVID-19, such as headache, olfactory and gustatory disorders, dizziness, confusion, impaired consciousness, epileptic seizures, cognitive dysfunction, coma, and cerebrovascular events [2,3,4,5]. Further research explored the possible pathophysiological pathways leading to neural cells invasion and the virus spreading within the Central Nervous System (CNS). Presently, the retrograde axonal transport via cranial nerves and transcytosis across the vascular endothelial cells of the Blood–brain barrier (BBB) seem to have the most solid scientific basis [6,7]. After entering the cell, the virus triggers various molecular pathways leading to dysregulation of the cell’s functioning or to its’ death via apoptosis, necroptosis, or autophagy. Additionally, direct cytopathic effects are possible [8]. On a tissue level, the virus causes damage by inducing inflammation (cytokine and immune cells related pathways), disrupting physiological regulatory systems (e.g., Renin-Angiotensin-Aldosterone axis), or destroying the intercellular connections. Direct viral neurotoxicity and tissue inflammation are believed to be the leading mechanisms responsible for nerve tissue damage [9].

Realizing that SARS-CoV-2 can infiltrate the patient’s nervous system, physicians began looking for diagnostic tools to assess the consequences of the CNS invasion. In living patients, Electroencephalography (EEG) and Magnetic Resonance Imaging (MRI) proved to be the most valuable solutions in this regard. 

As an electrophysiological study, the EEG examination of patients diagnosed with COVID-19 revealed disruptions in the functioning of the CNS’ neuronal networks. Epileptiform changes, slowing of background activity (both generalized and focal), and periodic discharges (e.g., sharp waves discharges) were amongst the most frequently reported EEG pathologies [10,11,12,13,14,15]. Unlike the functional changes, structural disorders associated with SARS-CoV-2 infection were best assessed using an MRI technique [16,17,18,19,20]. The most common MRI findings were acute ischemic or hemorrhagic changes within the brain, including microinfarcts [16]. Other findings comprised leukoencephalopathy, abnormal temporal lobe signals, contrast enhancement of the pia mater, and hyperintensity of the hippocampus [17,18,19,20].

Another helpful test in COVID-19 diagnostics is blood morphology. Leukogram alterations are a constant feature of any infection, SARS-CoV-2 being no different. Neutrophil/Lymphocyte Ratio (NLR) proved to be the most reliable predictor of COVID-19 severity and patient’s prognosis [21,22,23,24,25]. As one of the key action mechanisms of the SARS-CoV-2 virus is inducing a cytokine storm [26,27] in the host’s body, inflammatory markers are also widely used in patients with COVID-19. Serum C-reactive Protein (CRP) and Interleukin 6 (IL-6) are reliable predictors of disease severity and clinical outcomes [28,29,30].

A considerable issue with interpreting the additional tests’ results in COVID-19 is the secondary effects of severe hypoxia [31]. As brain tissue is very susceptible to oxygen shortage, hypoxia can induce both functional and structural changes in the CNS. EEG abnormalities in hypoxic patients were similar to those reported in patients with SARS-CoV-2 infection [32,33]. MRI findings in severe COVID-19 cases were attributed to brain tissue hypoxia [34,35]. For this reason, the patients included in this investigation were all classified as mild to moderate COVID-19 cases (more information in the Materials and Methods section). 

In this study, the authors combine data from patient’s medical history, physical examination, and additional test results in pursuit of any clinically relevant connections. The main objective of this study was to characterize the non-severe COVID-19 symptoms (especially neurological ones) and to confront them with additional test results in search of possible dependencies between the two.

## 2. Materials and Methods

Fifty-eight adult patients hospitalized in the Department of Neurology and Infectiology over a five-months period were retrospectively enrolled in this study. Due to the retrospective character of the study and full personal data confidentiality, no bioethical committee approval was obtained.

Considering the profile of the department in which this study was conducted, incoming patients could not be perceived as a random sample of the general patient population. Patients with suspected acute cerebral stroke or neurological symptoms were preferably referred to this Department of Neurology and Infectiology. In light of this fact, the authors abstained from assessing the coincidence of SARS-CoV-2 infection with cerebrovascular events in this study population. 

On admission, all patients included in this study were classified as mild or moderate COVID-19 cases, according to the World Health Organization (WHO) guidelines [36] (respiratory rate <30/min and oxygen saturation ≥90% on room air). None of these patients suffered from considerable hypoxia when additional tests (MRI, EEG) were performed, and the medical interview was taken. All data gathered in this investigation came from the time between the onset of the first symptoms (medical interview) to the fifth hospitalization day (presented symptoms and additional test results). By assuming such strategy, the authors avoided a secondary influence of severe hypoxia and hospitalization-associated complications on the study results. Each patient’s medical history was carefully reviewed before including him or her in the study population. Only patients with a complete medical interview regarding COVID-19 were enrolled. As mentioned above, every enrolled patient had to meet the non-severe SARS-CoV-2 infection criteria on admission. The medical history had to include information regarding the time of the first symptoms’ onset and the type of symptoms that the patient exhibited. 

Inclusion criteria:

- A positive COVID-19 diagnosis (based on the Real-Time Polymerase Chain Reaction test—95% cases; or based on clinical picture and anti-SARS-CoV-2 antibodies detection—5% cases).
- Non-severe COVID-19 on admission.
- A complete medical interview regarding COVID-19 infection.

Exclusion criteria:

- Severe COVID-19 on admission.
- An incomplete medical interview regarding COVID-19 infection.

Venous blood samples were taken, and a Computed Tomography (CT) scan of the chest was performed upon the admission of each patient. The following parameters of the venous blood were analyzed in this study: complete blood count, CRP, and IL-6. The NLR value was calculated retrospectively for each patient. Only the laboratory tests performed on admission were taken into consideration in this investigation. As patients reported to the hospital after various times from the symptoms’ onset, NRL, CRP, and IL-6 were compared in patients who reported to the hospital within a week from the symptoms’ onset and in those who reported later.

The following signs and symptoms were recorded in the enrolled patients: dyspnea, cough, fever, muscle aches, headache, fatigue, olfactory or gustatory disorders, and diarrhea. The exact date of the symptoms’ onset was also recorded. 

Chest CT was screened for COVID-19-specific lesions, such as ground-glass opacities, parenchyma consolidation, and septal thickening [37,38]. SARS-CoV-2-associated interstitial pneumonia was diagnosed based on clinical symptoms and CT findings. 

EEG was routinely performed in all patients within five days of admission as a part of an internal department’s protocol developed during the COVID-19 pandemic. 

EEG recordings were made using Elmiko DigiTrack technology (Elmiko, 05-822 Milanówek, Poland). The EEG protocol comprised 15 min of standard 19-channel EEG registration (electrodes: two pre-frontal, five frontal, four temporal, three central, three parietal, and two occipital). If the EEG technician noticed any abnormalities, the registration was prolonged by an additional 10–15 min. Three activations were used in this EEG protocol: hyperventilation (3 min), intermittent photic stimulation (2 min with closed eyes, using white light of 1–30 Hz frequency), and eye closure activation. EEG recordings were analyzed by an experienced physician, a specialist in the field of neurology. Patients with a previous medical history of epilepsy were excluded from further EEG analysis, as well as patients with acute ischemic/hemorrhagic lesions that corresponded with focal EEG abnormalities.

An MRI scan of the cerebrum was performed within five days of admission. Indications for this study included acute cerebral stroke, epileptic seizures in patients with no history of epilepsy, and persistent headaches. MRI images were analyzed by a team of two physicians, a radiology resident, and an experienced specialist in the field of radiology. 

Based on the literature, the following EEG [10,11,13] and MRI [16,17,19] features were recorded and analyzed in this study:

- EEG features:
  - Diffused background activity slowing.
  - Diffused background activity slowing.
  - Focal background activity slowing.
  - Generalized epileptiform discharges.
  - Focal epileptiform discharges.
  - Epileptic seizure/status epilepticus during the study.
  - Diffused sharp waves discharges.
- MRI features:
  - Acute/subacute cerebral stroke (hyperintense foci on T2/FLAIR images, with diffusion restriction).
  - Microhemorrhages (focal areas of signal loss on SWI images).
  - Abnormal temporal lobe signals (increased signal of the temporal lobe on T2 images).
  - Acute Disseminated Encephalomyelitis (ADEM) and other acute non-ischemic leasions.
  - Increased signal of the pia mater (on FLAIR images).
  - Abnormal hippocampi signal (increased signal of the hippocampi on FLAIR images).
  - White matter abnormalities (hyperintense foci on T2/FLAIR images, meeting the Fazekas scale 1, 2, or 3 criteria).
  - Acute/subacute intracerebral hematoma.
  - Cytotoxic Lesions of the Corpus Callosum (CLOCCs).

In the descriptive statistics of the quantitative variables, the following parameters were used: Median (M), interquartile range (Q1–Q3), mean, Standard Deviation (SD), and minimal and maximal values. The W Shapiro–Wilk test was used to assess the normality of the continuous variables’ distribution. The Mann–Whitney U test was used to compare the quantitative data between the groups; qualitative data were compared using the Chi2 test. The Spearman R correlation coefficient was used for correlation analysis. The Odds Ratio (OR) was calculated for predictive values assessment. A *p*-value of <0.05 was deemed statistically significant. Statistical calculations were performed with a licensed Statistica program.

## 3. Results

**Figure 1:**
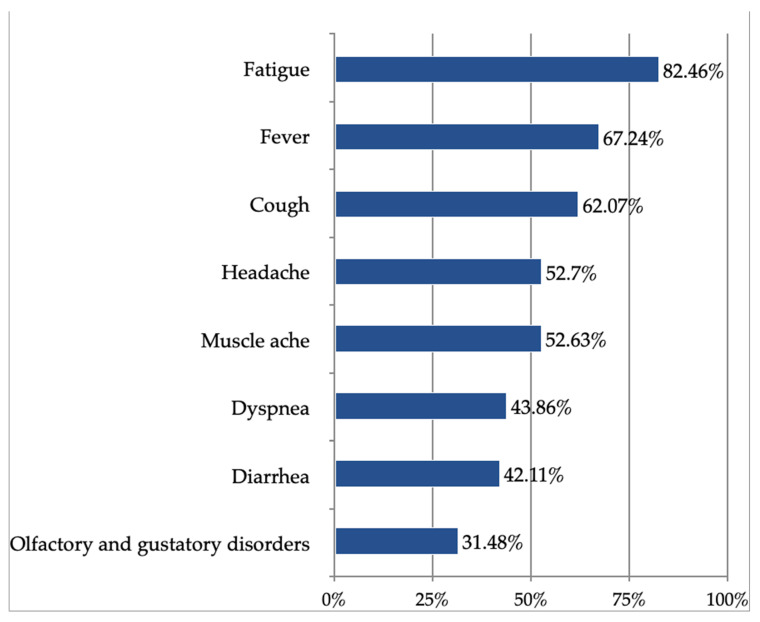
Signs and symptoms of the SARS-CoV-2 infection in the study population.

Fifty-eight adult patients reported to the hospital after the median of 6 (3–9.25) days from the symptoms’ onset. This population comprised 34 women (58.62%; mean age 58 ± 13 years) and 24 men (41.38%; mean age 64 ± 12 years). The most common signs and symptoms in these patients are presented in Figure 1. 

The following symptoms occurred statistically more often in females: cough (76.47% vs. 41.67% in males, *p* = 0.007), fatigue (93.94% vs. 66.67% in males, p=0.007), and headache (71.88% vs. 26.09% in males, *p* < 0.001). There was no statistically significant difference in the incidence of other symptoms between the genders. 

There was no statistically significant difference in NLR, CRP, and IL-6 between the genders (*p* = 0.266, *p* = 0.575, *p* = 0.314, respectively). There was no statistically significant difference in NLR, CRP, and IL-6 in patients who reported to the hospital within a week from the symptoms’ onset and in those who reported to the hospital later (*p* = 0.921, *p* = 0.562, *p* = 0.417, respectively). The NLR proved to be significantly higher in patients with abnormal EEG recordings (5.59 ± 6.11 vs. 2.96 ± 1.46 in the control group, *p* = 0.022).

Based on the chest CT scan and the clinical symptoms, SARS-CoV-2 associated pneumonia was diagnosed in 51 patients (87.93% of the study population).

**Figure 2:**
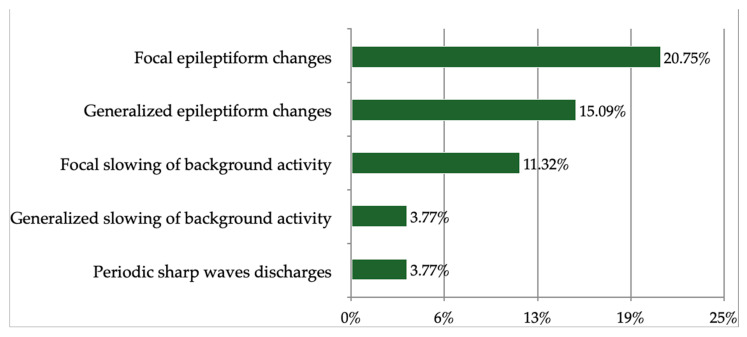
Abnormalities recorded in the EEG examination in the study population.

Eventually, 53 patients (91.37% of the study population) were included in further EEG analysis. Amongst them, 49.06% exhibited abnormal brain activity in the EEG examination. No patient had an epileptic seizure during the study. The abnormalities of the EEG recording are presented in Figure 2.

EEG abnormality occurrence did not correlate with the incidence of mild neurological symptoms (headache, olfactory, and gustatory disorders) of the SARS-CoV-2 infection. Similarly, there was no correlation between the incidence of these symptoms and MRI abnormalities occurrence—Table 1.

**Figure 3:**
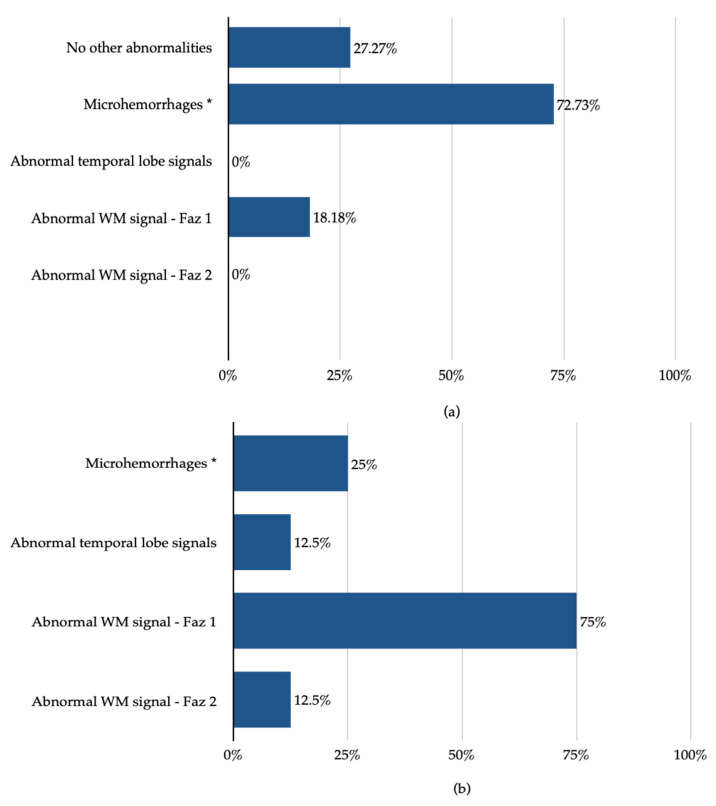
MRI findings in the study population: (**a**) MRI study in patients with acute cerebral stroke, (**b**) MRI study in patients without acute cerebral stroke. Legend: Faz 1—hyperintense white matter foci on T2/FLAIR images meeting the Fazekas scale 1 criteria, Faz 2—hyperintense white matter foci on T2/FLAIR images meeting the Fazekas scale 2 criteria, WM—white matter. *—the incidence of this finding differed significantly (*p* < 0.05) between patients with and without acute cerebral stroke.

An MRI study of the brain was performed in 26 patients (45% of the study population). Abnormalities were observed in 19 patients, of which 42.11% were pathologies associated with COVID-19, 15.78% were isolated acute ischemic/hemorrhagic lesions, and in the remaining 42.11% the above two coincided. All in all, COVID-19-associated pathologies were found in 27.59% of this study population. The abnormalities associated with SARS-CoV-2 infection are presented separately for patients with and without acute cerebral stroke—Figure 3. Microhemorrhages were found significantly more often in patients with acute cerebral stroke (72.73% vs. 25% in patients without acute cerebral stroke, *p* = 0.025).

EEG abnormalities were recorded in 9 patients with abnormal MRI findings other than or concomitant with acute cerebral stroke (56.25% vs. 30% in patients with no COVID-19-associated MRI findings, *p* = 0.147). The EEG abnormalities recorded in this group were focal epileptiform changes (66.67%), focal slowing of background activity (33.33%), and generalized epileptiform changes (11.11%).

In three patients in this study population, unprovoked generalized epileptic seizures occurred for the first time in their life. In all three cases, there were no acute cerebral lesions found in the head CT scan that could explain their symptoms. None of these patients required oxygen supplementation throughout their hospital stay. In one patient, the diagnosis of epilepsy was made as the seizures persisted during hospitalization. In the other two patients, no conclusive diagnosis could be made.

## 4. Discussion

According to the European Centre for Disease Control and Prevention (ECDC), most COVID-19 cases can be classified as mild [39]. These patients usually do not require hospitalization, and their prognosis for recovery is good. However, recent studies demonstrated that complications can occur even in mild COVID-19 cases [40,41], and long-lasting side effects of infection can persist [42,43]. For this reason, this investigation is focused on mild-to-moderate COVID-19 cases in terms of neurological manifestations of this disease. The authors aim to describe functional and structural changes within the brain in patients enrolled in this study, and, also, to correlate these changes with the patients’ symptoms.

It was demonstrated in this study population that women might be more prone to exhibit some of the general symptoms of SARS-CoV-2 infection. In this case, it was a cough, fatigue, and headache. Different researchers reached contradictory conclusions on this subject. Su et al. [44] in their study on 398 Taiwan patients concluded that there are no gender-based differences in COVID-19 symptoms, while Ahmed et al. [45] in their study on 440 Egyptian patients found that women were four times more likely to complain of cough. These differences may originate from different geographies (other SARS-CoV-2 variants present) or ethnicities [46].

Another finding of this study is a lack of significant difference between the CRP, IL-6, and NLR value in patients who reported to the hospital within a week from the symptoms’ onset and those who reported to the hospital later. The course of SARS-CoV-2 infection (similar to any infection) may vary between individuals. There are also sociological and psychological factors that influence the patient’s decision to seek medical attention. In light of the results of this study, the time from symptoms’ onset to reporting to the medical facility does not influence the prognostic value of CRP, IL-6, and NLR [21,30] in initially non-severe COVID-19 patients.

The incidence of COVID-19-associated chest CT findings in this study (87.93%) is similar to that reported by other authors [47]. Together with the fact that a CT scan was performed on admission when all patients were classified as non-severe COVID-19, it proves that lung tissue pathologies are very common even in patients with mild-to-moderate SARS-CoV-2 infection.

Many researchers report that EEG abnormalities are a common feature of COVID-19^⁠^ [11,15,48], which is consistent with this study’s results. However, many studies describing EEG abnormalities concern patients with severe COVID-19 [12,49] (often hospitalized in the ICU) or includes all grades of illness [15,50,51]. In this investigation, all the data comes from non-severe COVID-19 patients, thus eliminating the influence of severe brain tissue hypoxia. EEG abnormalities recorded in these patients can be attributed solely to SARS-CoV-2 infection, with only a minimal risk of other factors’ involvement. This is important, as medical professionals caring for COVID-19 patients need to be aware that even non-severe SARS-CoV-2 infection can cause functional changes within the CNS. The affinity of novel Coronavirus to nerve tissue is well established [2,6]. Neuroinflammation and cytotoxic effects mediated by proinflammatory agents are believed to be the main factors responsible for neurological impairment in COVID-19⁠ [52,53,54]. This research is also consistent with this investigation’s finding that EEG abnormalities were significantly associated with higher NLR. This indicates that NLR may have a broader application in COVID-19 management than predicting the disease’s severity.

Similar to EEG abnormalities, MRI findings in patients with SARS-CoV-2 infection are often described in populations involving both non-severe and severe cases [16,17,51]. MRI findings in this non-severe COVID-19 patient population are consistent with those reported by other authors [16,17,19,20]. In this research, the authors abstained from investigating the frequency of the gravest MRI finding—acute cerebral stroke—for reasons described in the Materials and Methods section. However, a significantly greater incidence of brain microhemorrhages was observed in patients with acute cerebral stroke in this study population. This observation is baffling for two reasons. Firstly, mean microhemorrhages’ incidence in patients with acute cerebral stroke is estimated to be 16–38%, according to a recent meta-analysis by Charidimou et al. [55], whereas in this study population their incidence reached 72.73%. Secondly, brain microhemorrhages are usually observed in critically ill patients with COVID-19, and many authors speculate whether their presence is related to viral infection or to hypoxia and cytokine storms in these patients [56,57,58,59]. Considering the modest sample size of this study, the authors confine to concluding that brain microhemorrhages can also be found in non-severe COVID-19 patients, and their association with acute cerebral stroke requires further investigation.

An attempt was made in this investigation to correlate the incidence of mild neurological symptoms (headache, gustatory, and olfactory disorders) suggestive of some CNS disorders with abnormalities in EEG and MRI examination. Statistical analysis revealed no significant association between these two. Headache as well as gustatory and olfactory disorders do not indicate the presence of either EEG or MRI abnormalities in COVID-19. In a group of patients with abnormal MRI findings, there was a high percentage of EEG abnormalities. The incidence of abnormal EEG recordings did not differ significantly between patients with and without COVID-19-associated MRI findings. Nevertheless, over half of the patients with COVID-19-associated MRI findings had abnormal EEG recordings, which proves that functional and structural changes of the brain frequently coincide with one another in SARS-CoV-2 infection. To the best of this study’s authors’ knowledge, the literature on this particular subject is scarce [60].

In three patients in this study population, first-time unprovoked generalized epileptic seizures occurred. In one patient, it proved to be the onset of epilepsy, while it was a one-time episode in the other two cases. Scientific literature is consistent about the fact that SARS-CoV-2 infection can be a trigger factor for seizures, both in epileptic and non-epileptic patients [61,62,63]. Yet, again, the research on this subject comprises mainly the general COVID-19 population (severe and non-severe cases) or solely severe and critical cases [64]. The present investigation demonstrates that non-severe COVID-19 can also be a trigger factor for epilepsy and epileptic seizures.

It is worth mentioning that, according to the local epidemiological situation at the time of the study assessed by Serwin et al. [65], the patients in this study population were most probably infected with the alpha variant of the SARS-CoV-2 virus. Presently, other SARS-CoV-2 variants that mutated from the alpha variant are predominant in the general population. Nevertheless, symptoms of the infection (neurological ones included) did not change significantly [66,67], thus leaving this research up to date with the current epidemiological situation.

This study had several limitations. The mayor limitation of this study is the modest sample size. This is due to the fact that many patients had incomplete medical interviews regarding their SARS-CoV-2 infection, which was the main cause for exclusion. One may argue that this creates a bias, as the test results of patients with incomplete medical histories were disregarded. During the study enrollment, the authors decided that high data quality is a priority and outweighs the possible hazard of selection bias. Another limiting factor in this investigation was the percentage of patients who had the brain MRI performed, as the conclusions regarding combined EEG and MRI results had to be drawn from a small patient group.

## 5. Conclusions

Non-severe SARS-CoV-2 infection causes functional and structural abnormalities within the central nervous system. Brain microhemorrhages are frequently present in non-severe COVID-19 patients. There is no significant association between mild neurological symptoms of COVID-19 and additional test abnormalities. The time from SARS-CoV-2 infection’s onset to hospital admission does not seem to influence the prognostic value of CRP, IL-6, and NLR in non-severe COVID-19. Mild-to-moderate SARS-CoV-2 infection can be a trigger factor for epilepsy and epileptic seizures.

## Figures and Tables

**Table 1:** The odds ratio of mild neurological symptoms coinciding with EEG and MRI abnormalities.

	EEG Abnormalities			
Independent variable	OR	−95% CI	+95% CI	*p*
Headache	1.75	0.58	5.32	0.324
Olfactory and gustatory disorders	0.65	0.20	2.15	0.486
	MRI abnormalities			
Independent variable	OR	−95% CI	+95% CI	*p*
Headache	2.00	0.45	8.98	0.366
Olfactory and gustatory disorders	1.11	0.24	5.08	0.896

Legend: CI—confidence interval, OR—odds ratio, *p*—*p*-value.

## Data Availability

The data presented in this study are available on request from the corresponding author. The data are not publicly available due to confidentiality reasons.
